# Evaluation of mandibular lingula and foramen location using 3-dimensional mandible models reconstructed by cone-beam computed tomography

**DOI:** 10.1186/s40902-017-0128-y

**Published:** 2017-10-25

**Authors:** Cong Zhou, Tae-Hyun Jeon, Sang-Ho Jun, Jong-Jin Kwon

**Affiliations:** 1 Division of Oral & Maxillofacial Surgery, Department of Dentistry, Korea University Anam Hospital, 73, Inchon-ro, Seongbuk-gu, Seoul, 136-705 South Korea; 2 School of Stomatology, Stomatological Hospital of Shandong University, #44-1, Lixia District, Wenhuaxi Road, Jinan City, Shandong Province China; 3 Shandong Provincial Key Laboratory of Oral Tissue Regeneration, Jinan, China; 4 Korea University, Seoul, South Korea

**Keywords:** 3D Anatomy, Mandibular ramus, Lingula, Foramen, Cone-beam CT

## Abstract

**Background:**

The positions of the mandibular lingula and foramen have been set as indexes for inferior alveolar nerve (IAN) block and ramus osteotomies in orthognathic surgery. This study aimed to evaluate the anatomical structures of mandibular ramus, especially the mandibular lingula and foramen, by analyzing the cone-beam computed tomography (CBCT) data of young adults.

**Methods:**

We evaluated 121 sides of hemi-mandibular CBCT model of 106 patients (51 male and 55 female patients; 18 to 36 years old). All the measurements were performed using the 2- and 3-dimensional rulers of OnDemand3D® software.

**Results:**

Statistical analysis of the data revealed that there was no significant difference in the mandibular angle between the genders. The mandibular lingula was found to be located at the center of ramus in males, but a little posterior in relation to the center in females. The mandibular lingula was rarely located below the occlusal plane; however, the position of the mandibular foramen was more variable (84.3% below, 12.4% above, and 3.3% at the level of the occlusal plane).

**Conclusions:**

The results of this study provide a valuable guideline for IAN block anesthesia and orthognathic surgery. CBCT can be considered effective and accurate in evaluating the fine structures of the mandible.

## Background

It is important to know the precise anatomical positions of the mandibular lingula (ML) and mandibular foramen (MF) in routine dental practice, especially during block anesthesia of inferior alveolar nerve (IAN) and orthognathic surgery. The failure rate of IAN block has been reported to range between 10 and 39% [1, 2], and the most common reason for this failure points to the inaccurate placement of the hypodermic needle tip, which is not close enough to the MF [3]. Proper evaluation of the anatomical landmarks in relation to the IAN, such as the MF and ML, is the key to the achievement of effective anesthesia of the IAN in clinical practices of the mandible. Dentofacial deformities, especially mandibular prognathism and retrognathism that are caused by abnormal growth of the jaw, occur in a relatively high incidence among Asians [4]. In recent years, sagittal split ramus osteotomy has become a routine surgical technique for the correction of these deformities owing to its advantages, such as the intraoral approach, easy internal fixation, decreased healing time, and early jaw function [5–7]. Determining the precise anatomical locations of the ML and MF is essential in order to achieve a favorable fracture line on the mandibular ramus and prevent IAN damage and other complications during orthognathic surgery. The ML has been described as an important surgical landmark for horizontal osteotomy in orthognathic surgery because the horizontal osteotomy is positioned close to the ML and IAN [5–8]. Further, the accurate location of the ML is critical to a large number of other oral and maxillofacial surgical procedures, such as mandibular trauma management, benign and malignant tumor removal, mandibular and temporomandibular joint (TMJ) reconstruction, and pre-prosthetic surgery [9].

The application of cone-beam computed tomography (CBCT) in dentistry has rapidly developed in recent years, especially in implantology, because CBCT has been shown to overcome many disadvantages of conventional medical computed tomography (CT), such as the high dose of radiation, long radiation exposure time, and low resolution ratio. It has been reported that the radiation dose of CBCT is just 25% of the radiation dose of a panoramic radiograph and 1.6 to 2.5% of that of a conventional medical CT [10, 11]. One of the most remarkable advantages of CBCT is its high resolution ratio; a voxel size as small as 0.125 mm can be achieved with CBCT, which translates into a powerful ability to obtain accurate 3-dimensional (3D) reconstructions [12–15].

Recently, CBCT has been used frequently to determine the accurate anatomy of oral and maxillofacial structures, such as the root canal system, inferior alveolar canal, impacted teeth, TMJ, and even the upper respiratory tract [15–23]. Therefore, this retrospective study is designed to use CBCT data to verify the positions of the MF and ML in relation to the surrounding landmarks; and to give an accurate description of the anatomical morphology of the mandibular ramus.

## Methods

This retrospective study was based on the CBCT data collected from the Division of Oral and Maxillofacial Surgery, Department of Dentistry, Korea University Anam Hospital, Seoul, between 4 June, 2013, and 29 July, 2014 (IRB approval: AN14291-001). Patients under 18 years of age were excluded from the study owing to the incomplete development of the mandible. Also, the patients with syndromic craniofacial deformity were excluded. Most of the patients had been advised to undergo CBCT scans of the mandibular body and ramus in order to determine the 3D relationship of the third molar with the inferior alveolar canal prior to its extraction.

All the CBCT examinations had been carried out using an AZ3000CT 3D imaging system (Asahi Roentgen Co., Kyoto, Japan). The imaging parameters had been set as follows: 6 mA, 85 kV, 0.5 × 0.5 mm fixed focal spot, and the field of view (FOV) of 80-mm height and 75-mm diameter. The total scanning time had been 17 s.

**Fig. 1:**
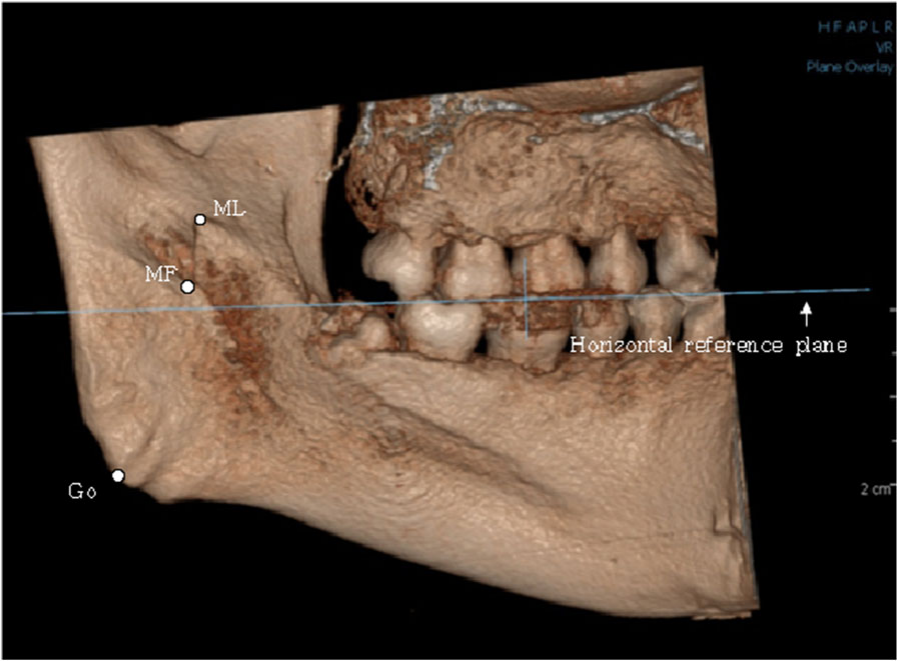
The occlusal plane was set as the horizontal reference plane to standardize the 3D positions of all the mandible models. (ML, mandibular lingula; MF, mandibular foreman; Go, gonion)

**Fig. 2:**
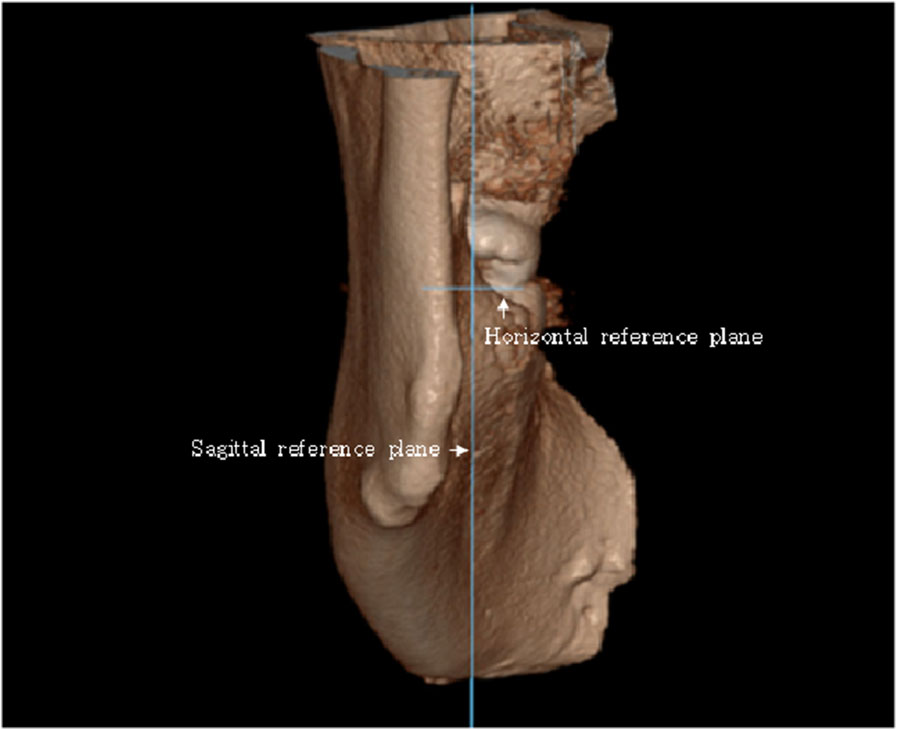
The plane perpendicular to the occlusal plane and along the line from the cusp of canine to the mesiobuccal cusp of the first molar was set as the sagittal reference plane

Using the CBCT data, 3D models of the mandibles were constructed using OnDemand3D® software (CyberMed International, Seoul, Korea) with a voxel size of 0.2 mm and slice thickness of 0.2 mm. Prior to obtaining measurements, two reference planes were defined to standardize the 3D positions of all the mandibular models. The occlusal plane was defined using three points (mesiobuccal cusp tips of both mandibular first molars and a mid-point of both mandibular central incisors) as the horizontal reference plane, and a plane along the line from the cusp of the canine to the mesiobuccal cusp of the first molar and perpendicular to the occlusal plane was defined as the sagittal reference plane (Figs. 1 and 2).

All measurements were carried out twice by the same operator with the mandibular models placed in a standardized position. Analysis was conducted using mean values of two repeated measurements and if two measurement values showed over 1-mm differences measured again and used two values having lower differences. The ML was set as the reference point for the measurements; measurements were obtained using the 3D ruler of OnDemand3D® software (Table 1). With the occlusal plane for reference, the anteroposterior and vertical relationships of the ML and MF were measured using the 2-dimensional (2D) ruler.

**Table 1:** Measurements of mandibular ramus morphology

1. Mandibular angle: angle between two tangent lines of ower border and posterior border
2. ML–MF: distance from ML to MF
3. AP: anteroposterior ramal dimension at ML parallel to OP
4. ML–internal line: distance from ML to internal oblique line parallel to OP
5. ML–external line: distance from ML to external oblique line parallel to OP
6. ML–posterior line: distance from ML to posterior border of the ramus parallel to OP
7. ML–SN: distance from ML to the lower point of sigmoid notch
8. ML–second molar: distance from ML to the CEJ of mandibular second molar
9. ML–lower border: distance from ML to the lower border of ramus
10. ML–Go: distance from ML to Go

Abbreviations: ML, mandibular lingula; MF, mandibular foreman; AP, anteroposterior; OP, occlusal plane; SN, sigmoid notch; Go, gonion

The statistical differences in the mandibular ramus morphology between male and female subjects were determined using independent *t*-tests with a significance level of *P* < 0.05. All the statistical analyses were performed using SPSS 21.0 (SPSS Inc., IL, USA).

## Results

In this study, 121 sides of hemi-mandibular CBCT models of 106 patients (51 male and 55 female patients; mean age 26.8 ± 8.7 years, range 18 to 36 years) were examined. Of these, 101 patients had undergone CBCT examination for determining the 3D relationship of the third molar with the inferior alveolar canal prior to its extraction; the remaining 5 patients had undergone this test in order for examination of cystic lesions in the molar region.

The results of the measurements of the mandibular ramus are presented in Table 2. The mean mandibular angle was 125.1° in males and 124.1° in females. No statistically significant difference in the mandibular angle based on gender was detected (*P* > 0.05). At the ML level, the anteroposterior ramal dimension was significantly greater in males (34.6 ± 2.4 mm) than in females (31.5 ± 2.4 mm) (*P* < 0.05). In males, the ML was found to be located in the center of the width of the ramus, with the same mean distance of 18.2 mm from the most anterior and posterior border of the ramus. However, it was positioned a little posterior to the center of the ramus in females, with a mean distance of 18.3 ± 2.2 mm from the anterior border and 17.0 ± 1.8 mm from the posterior border of the ramus.

**Table 2:** Data of measurements on mandibular ramus morphology

Measurement	Sex	Mean ± SD	Min~Max	*P* value
Mandibular angle (°)	M	125.1 ± 4.9	112.3~134.9	0.240
	F	124.1 ± 4.9	108.3~134.0	
ML–MF (mm)	M	10.1 ± 2.3	4.8~15.9	0.385
	F	9.8 ± 2.1	5.4~15.7	
AP (mm)	M	34.6 ± 2.4	29.4~39.6	0.014*
	F	31.5 ± 2.4	29.2~39.8	
ML–internal line (mm)	M	13.9 ± 1.9	10.7~19.4	0.424
	F	13.6 ± 2.1	8.6~17.7	
ML–external line (mm)	M	18.2 ± 2.4	14.3~25.6	0.894
	F	18.3 ± 2.2	12.8~24.1	
ML–posterior line (mm)	M	18.2 ± 1.7	15.2~21.7	0.000*
	F	17.0 ± 1.8	12.1~21.4	
ML–SN (mm)	M	15.7 ± 2.7	11.1~23.1	0.768
	F	15.5 ± 2.3	11.7~22.9	
ML–second molar (mm)	M	31.0 ± 3.3	21.6~36.3	0.001*
	F	28.1 ± 2.9	24.4~37.4	
ML–lower border (mm)	M	35.3 ± 3.3	27.0~42.8	0.000*
	F	30.5 ± 2.8	25.2~38.5	
ML–Go (mm)	M	33.8 ± 3.2	24.5~41.9	0.000*
	F	28.9 ± 3.0	22.7~35.9	

Abbreviations: ML, mandibular lingula; MF, mandibular foreman; AP, anteroposterior; SN, sigmoid notch; Go, gonion

*Statistical significance

The distance of the ML from the lower border of mandible (35.3 ± 3.3 mm in males; 30.5 ± 2.8 mm in females) was always greater than its distance from the mandibular sigmoid notch (15.7 ± 2.7 mm in males; 15.5 ± 2.3 mm in females). The ML was located approximately at the junction of the upper one third and lower two thirds of the line joining the lower border of the ramus and the sigmoid notch. The distance of the ML from the CEJ of the second molar in males (31.0 ± 3.3 mm) was found to be statistically greater than that in females (28.1 ± 2.9 mm) with *P* < 0.05. The data showed a statistically significant difference between males and females in relation to the distance of the ML from the gonion (*P* < 0.05); the ML was farther from the gonion in males.

**Fig. 3:**
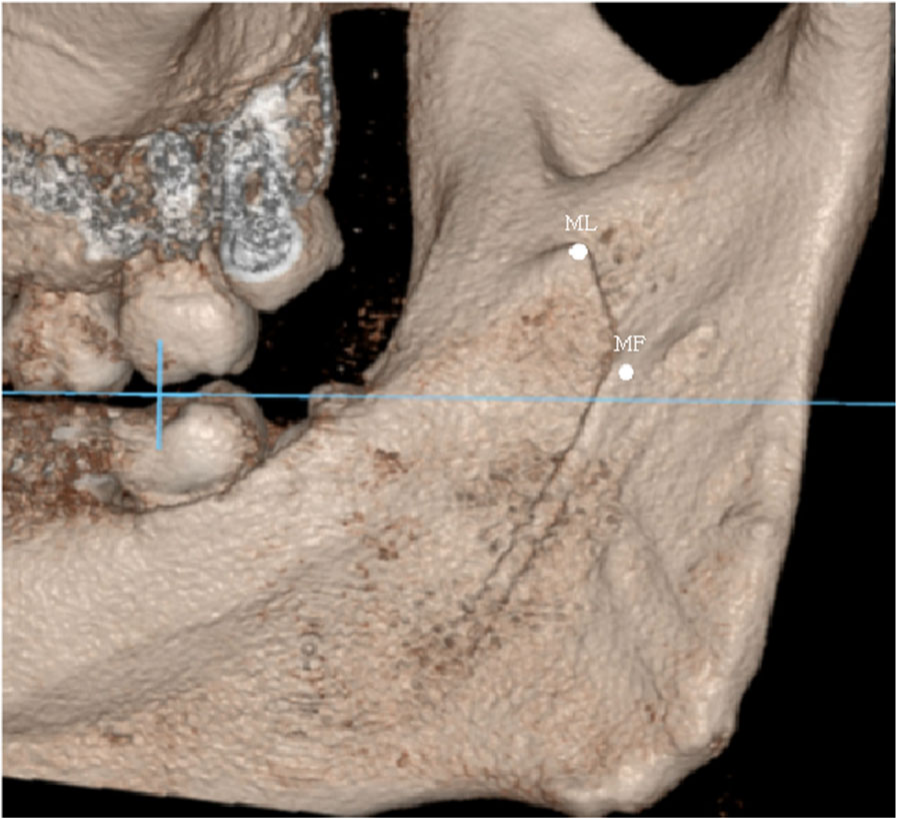
Both of the ML and MF above the occlusal plane; ML in front of MF. The blue line indicates the occlusal plane

**Fig. 4:**
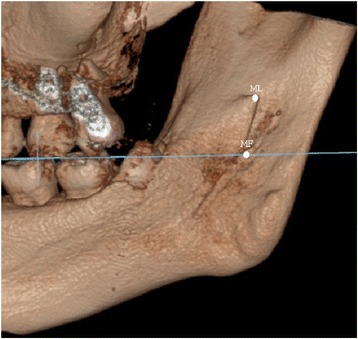
ML above the occlusal plane; MF at the level of occlusal plane. The blue line indicates the occlusal plane

**Fig. 5:**
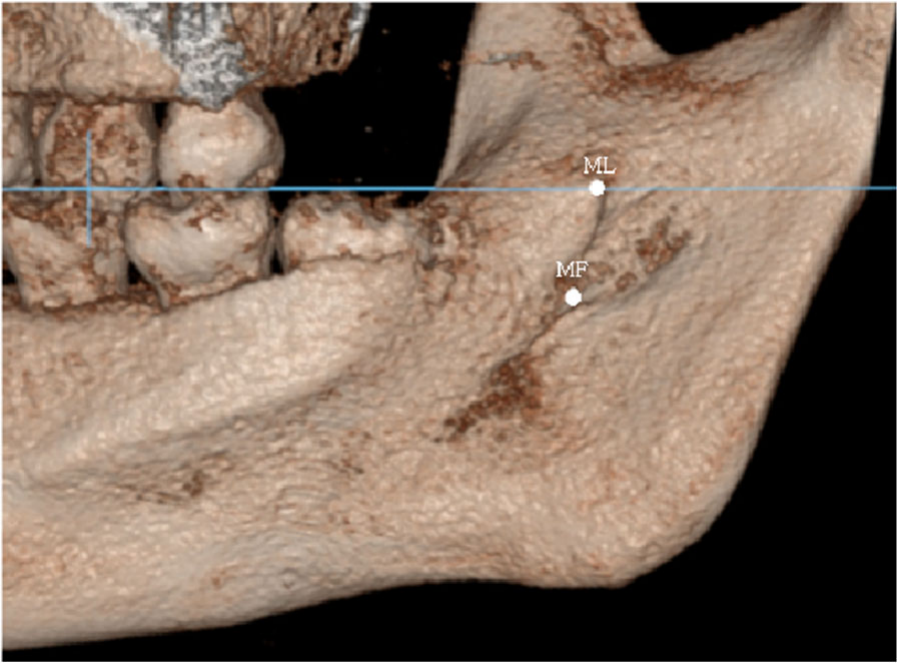
ML at the level of occlusal plane; MF below the occlusal plane; MF in front of ML. The blue line indicates the occlusal plane

The data revealed great variation in the 3D distance between ML and MF (4.8 to 15.9 mm in males; 5.4 to 15.7 mm in females). Further, the anatomical forms of the ML and MF displayed great variability, too. In the standard position, the relationship of the ML and MF to the occlusal plane was evaluated. We found that 98.3% of the lingulae were located 6.0 ± 2.9 mm above the occlusal plane (Figs. 3 and 4); one ML (0.8%) was equal with the occlusal plane (Fig. 5) and one (0.8%) was below the occlusal plane (Table 3).

**Table 3:** Relationship of mandibular lingula to occlusal plane

Sex	Above		Equal	Below		Total	
	No.	Mean ± SD (mm)	No.	No.	Mean ± SD (mm)	No.	Mean ± SD (mm)
Male	58	6.2 ± 2.8	0	0	0	58	6.2 ± 2.8
Female	61	5.8 ± 2.9	1	1	-1.9	63	5.6 ± 3.1
Total	119 (98.3%)	6.0 ± 2.9	1 (0.8%)	1 (0.8%)	-1.9	121 (100%)	5.9 ± 3.0

Abbreviations: No., number of sides; MD, mean distance from mandibular lingula to occlusal plane

With regard to the MF, it was found that 84.3% of the foramina were inferior to the occlusal plane with a mean distance of 4.5 ± 2.6 mm (Fig. 5); 12.4% were located 2.5 ± 2.3 mm above the occlusal plane (Fig. 3); and only 3.3% were equal with the occlusal plane (Fig. 4; Table 4).

**Table 4:** Relationship of mandibular foramen to occlusal plane

Sex	Above		Equal	Below		Total	
	No.	Mean ± SD (mm)	No.	No.	Mean ± SD (mm)	No.	Mean ± SD (mm)
Male	5	3.5 ± 3.4	3	50	-4.2 ± 2.3	58	-3.2 ± 3.3
Female	10	2.1 ± 1.6	1	52	-4.8 ± 2.9	63	-3.6 ± 3.7
Total	15 (12.4%)	2.5 ± 2.3	4 (3.3%)	102 (84.3%)	-4.5 ± 2.6	121 (100%)	-3.4 ± 3.5

Abbreviations: No., number of sides; MD, mean distance from mandibular lingula to occlusal plane

The anteroposterior relationship of the ML and MF on the occlusal plane was evaluated, too. The MF was primarily located 2.6 ± 1.7 mm in front of the ML (75.2%; Fig. 5), although sometimes it was located 1.4 ± 0.7 mm behind the ML (17.4%; Fig. 3); the line connecting the MF and ML was rarely perpendicular to the occlusal plane (7.4%; Table 5).

**Table 5:** Anteroposterior relationship of ML and MF on occlusal plane

Sex	MF in front of ML		Line connecting MF and ML perpendicular to OP	ML in front of MF	
	No.	Mean ± SD (mm)	No.	No.	Mean ± SD (mm)
Male	46	2.5 ± 1.8	2	10	1.6 ± 0.5
Female	45	2.7 ± 1.6	7	11	1.2 ± 0.8
Total	91 (75.2%)	2.6 ± 1.7	9 (7.4%)	21 (17.4%)	1.4 ± 0.7

Abbreviations: ML, mandibular lingula; MF, mandibular foreman; OP, occlusal plane; No., number of sides; MD, mean distance from ML to MF on OP

## Discussion

During the procedures of block anesthesia of IAN and orthognathic surgery, it is important to locate the ML and MF accurately. Nevertheless, there is still some disagreement in the anatomical description of the mandibular ramus, especially in relation to the ML and MF. Thus far, the available anatomical data on the mandibular ramus has mostly been based on the measurements of dry human skulls. In most cases, however, dry human skulls cannot adequately provide the data on sex, age, or race due to lack of information [6]. This study mainly included patients 20 to 30 years of age. However, most of the patients undergoing third molar extraction and orthognathic surgery are around 20 to 30 years of age, it is likely that this age distribution is meaningful.

CBCT, in contrast to conventional CT, offers higher resolution with lower radiation exposure [10–15]. The accuracy of 3D measurement is influenced by the slice thickness and voxel size. The slice thickness of the CBCT used in this study was 0.2 mm and the voxel size was 0.2 mm. Therefore, the accuracy of the 3D images reconstructed in this study can be considered acceptable.

In cosmetic surgery, the mandibular angle is identified as an important indicator in the evaluation of the shape of the face [24]. In an earlier study, Hetson et al. [25] measured the mandibular angle on 317 hemisected dried human mandibles using a precisely designed photographic technology and found the mean mandibular angle to be 123°. After measuring 60 panoramic radiographs, Pirgousis et al. [26] reported that the mean mandibular angle was 123.6° in females and 123.43° in males with no significant difference between the genders. This corroborates the result of the present study. Depending on our study conducted on young Koreans, mean mandibular angle was 125.1° in females and 124.1° in males. The distances of the ML from the mandibular second molar, lower border of the mandible, and the angle of the mandible were found to be statistically greater in males than in females. However, we did not find statistically significant differences between males and females in relation to the distance of the ML from the sigmoid notch. It can therefore be concluded that the segment of the mandibular ramus below the ML may be bigger in males than in females.

As a surgical reference point in orthognathic surgery, prior to performing the medial horizontal osteotomy, the ML must be located in order to maintain a safe distance of at least 5 mm from the MF [27]. The positions of ML and MF have been reported in many studies; however, the results are variable. After measuring the panoramic radiographs of 73 Thai adult mandibles, Kositbowornchai et al. [9] found that the ML was located posterior to the center of the width of the ramus and the MF was much closer to the sigmoid notch than to the lower border of mandible. Nicholson [28] measured 80 dry adult mandibles of East Indian ethnic origin and reported that the foramen was exactly halfway between the mandibular notch and the inferior border of mandible in the upper third of the line connecting the coronoid process with gonion. In this study, it was found that the ML was located at the center of the width of the ramus in males and slightly posterior to the center of the ramus in females. In the vertical direction, the ML was found at the junction of the upper one third and lower two thirds of the line joining the lower border of the ramus and the sigmoid notch. The mean distance of ML to the occlusal plane was 5.9 mm above the occlusal plane, which could be a valuable indicator for locating the ML during orthognathic surgery. The present study indicated that 75.2% of the foramina were located in the front of the ML when the occlusal plane was set as the reference plane. However, in a previous study, Hayward et al. [29] reported the MF was located just posterior to the ML. This difference can probably be explained by the different reference points and planes used during the measurement procedure. Also, we measured distance from ML to Go. In our study, mean distance was 28.9 mm in females and 33.8 mm in males. Above information could help approximatively estimate inferior alveolar nerve position in orthognathic surgery on gonion area such as mandibular angle reduction.

If the relationship between MF and occlusal plane can be confirmed, it will be much easier to achieve successful IAN block anesthesia. Nicholson [28] reported that 75% of the foramina were below the occlusal plane and 22.5% of them leveled with the occlusal plane. After studying 38 dry mandibles of adult black Zimbabweans, Mbajiorgu [30] found that 47.1% of the foramina leveled with the occlusal plane and 29.4% were above the occlusal plane. In the present study, we found that 84.3% of the foramina were 4.5 mm below the occlusal plane. In contrast, Kositbowornchai et al. [9] found that the MF was 10 mm above the occlusal plane in their study using panoramic radiographs. Since there is a great degree of variability regarding the position of the MF, it is difficult to define the accurate needling position and depth during the IAN block.

## Conclusion

Depending on our study, we found that 84.3% of the mandibular foramina were 4.5 mm below the occlusal plane. Also, the mean distance of mandibular lingula to the occlusal plane was 5.9 mm above the occlusal plane. Above information as anatomical indications may be helpful for block anesthesia of inferior alveolar nerve (IAN) and orthognathic surgery.
